# How Do Addressees Exploit Conventionalizations? From a Negative Reference to an *ad hoc* Implicature

**DOI:** 10.3389/fpsyg.2019.01461

**Published:** 2019-07-03

**Authors:** Edmundo Kronmüller, Ira Noveck

**Affiliations:** ^1^Escuela de Psicología, Pontificia Universidad Católica de Chile, Santiago, Chile; ^2^Institut des Sciences Cognitives – Marc Jeannerod (UMR 5304), Lyon, France

**Keywords:** pragmatics, negation, reference, conventions, *ad hoc* implicature, eye-tracking

## Abstract

A negative reference, such as “not the sculpture” (where *the sculpture* is a name the speaker had only just invented to describe an unconventional-looking object and where the negation is saying that she does not currently desire that object), seems like a perilous and linguistically underdetermined way to point to another object, especially when there are three objects to choose from. To succeed, it obliges listeners to rely on contextual elements to determine which object the speaker has in mind. Prior work has shown that pragmatic inference-making plays a crucial role in such an interpretation process. When a negative reference leaves two candidate objects to choose from, listeners avoid an object that had been previously named, preferring instead an unconventional-looking object that had remained unnamed (Kronmüller et al., 2017). In the present study, we build over these findings by maintaining our focus on the two remaining objects (what we call the *second* and *third* objects) as we systematically vary two features. With respect to the second object – which is always unconventional looking – we vary whether or not it has been given a name. With respect to the third object – which is never named – we vary whether it is unconventional or conventional looking (for the latter, imagine an object that clearly resembles a bicycle). As revealed by selection patterns and eye-movements in a visual-world eye-tracking paradigm, we replicate our previous findings that show that participants choose randomly when both of the remaining objects are unconventional looking and unnamed and that they opt reliably in favor of the most nondescript (the unnamed unconventional looking) object when the second object is named. We show further that (unnamed) conventional-looking objects provide similar outcomes when juxtaposed with an unnamed unconventional object (participants prefer the most non-descript as opposed to the conventional-looking object). Nevertheless, effects emerging from the conventional (unnamed) case are not as strong as those found with respect to those reported when an unconventional object is named. In describing participants’ choices in the non-random cases, we propose that addressees rely on the construction of an *ad hoc* implicature that takes into account which object can be eliminated from consideration, given that the speaker did not explicitly name it.

## Introduction

**Figure 1:**
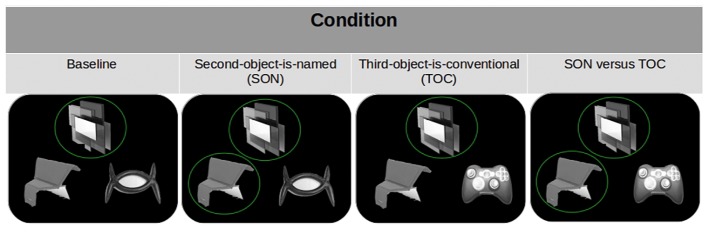
An example of a test trial screen in each condition whose final instruction is “not the sculpture.” These are, from left to right, the baseline, the second-object-named (SON), the third-object-conventional (TOC), and the SON-vs.-TOC conditions. The upper object in each is the negated reference that has been heretofore mentioned twice as “the sculpture.” A green circle indicates that the object (an unconventional one) has been given a name (circles do not appear on the participant’s screen). In these examples, the object on the lower left hand side is the second (Y) object that has been called “the bench” (twice until now) in the second-object-named (SON) condition as well as in the SON-vs.-TOC condition. The object on the lower right hand side is the third (Z) object; this object can be rendered conventional and readily identifiable (as a “gamepad”) in the TOC and SON-vs.-TOC conditions.

Imagine you are asked to assist a jewelry shop owner as she prepares a window display of newly arrived, hard-to-describe brooches. While you are at the storefront poised to display a brooch, the owner is sitting behind a computer screen while looking at two brooches at a time in order to make thoughtful comparisons. Over the course of her deliberation, the owner refers to the brooches by giving approximate names for them (e.g., the *ballerina*, the *insect*, etc.). You now pull out a box-set of three other hard-to-describe brooches from an up-and-coming jewelry-designer as the owner, again, ponders two at a time. While sharing her impressions, she soon refers to one of the three in the box as “the one that looks like a modern sculpture,” which you can now identify. While pondering over which one of the three she wants to display, she finally shouts out what we call a negative reference (Kronmüller et al., 2017) – “not the sculpture” – because she actually wants the other one on her screen. Which one is she referring to? Prior experimental work, which captures just such a situation in more austere conditions, has shown that when the speaker employs “not” in such a context, the addressee (the participant) will randomly choose from the remaining two under consideration (given that there is no possibility for the listener to ask the speaker which one). This makes sense given that, from the addressee’s perspective, there remain two possibilities out of three. Interestingly, the work on negative reference further shows that when two of the three objects have been given a name (to return to the scenario above, imagine one brooch has been coined the *sculpture* and a second one the *bench*), listeners will typically eliminate the second, named object from consideration as well (and reliably so, at a rate of about 80%) while ultimately pointing to the only remaining unnamed object (see Kronmüller et al., 2017). See Figure 1 for a representation of these two, critical (*baseline* and *second-object-is-named*) conditions. Such phenomena reveal that addressees assume that an interlocutor will use an agreed-upon name when it is available. The current work extends the prior work by employing this confirmed paradigm to investigate the case in which an unnamed object – one that could have a conventional name (e.g., imagine an iconic representation of a bicycle or a gamepad) – similarly determines performance on such a negative reference task.

In the remainder of the section “Introduction,” we will do three things. First, we will describe how the task reported in this study came into being, and we will present its features in greater detail so as to elucidate how prior studies have been useful in making claims about reference. Second, we will show how key results from this class of experiments can be viewed as *ad hoc* implicatures, as introduced in the scalar inference literature (Papafragou and Tantalou, 2004; Barner et al., 2011; Stiller et al., 2015). Finally, we will describe how we modified the task for the purposes of the present study in order to investigate the role of conventionality with respect to this pragmatic inference.

### Background

This particular paradigm actually evolved without negations to address a debate that aimed to answer the following questions: does a listener incorporate who named an (unusual looking) object when two different speakers are doing the naming independently, or does the listener treat the object name as author-free? Consider the case of a voice (heard over headphones) that helpfully identifies an unusual looking object for the participant by calling it “the thing that looks like a modern sculpture” and eventually just “the sculpture.” When a new voice also calls the object *the sculpture*, do participants’ looks go directly to the object (on a screen) without hesitation? Are participants surprised when the second voice (these tasks are usually carried out with distinctive male and female voices) comes up with a new name for an object? Prior work had led to multiple eye-tracking experiments with straightforward affirmative references (Barr and Keysar, 2002; Metzing and Brennan, 2003; Kronmüller and Barr, 2007; Brown-Schmidt, 2009), all designed to capture how immediately a person looks at and clicks on a previously named object when that object is referred to by a new speaker (this paradigm also detects how much confusion is produced when the same speaker changes an object’s previously given name). In a meta-analysis of this work, Kronmüller and Barr (2015) showed that participants find objects without fully paying heed to the source of the coinage; rather, they pay attention to the fact that an object has a name attached to it.

The negative reference study in Kronmüller et al. (2017), described above, was built to further explore the timing involved with respect to the source of coinage (by including two speakers) when resolving reference. In the present work, we aim to exploit this paradigm’s reliable findings when faced with a *solitary* speaker. Our goal is more fundamental, which is to determine whether a conventional-looking object that remains unnamed holds as much sway as an unconventional-looking object that has been referred to with an agreed-upon name. In this way, we can explore how two different kinds of conventions (one being the invented names briefly shared by interlocutors and the other being conventional representations that are presumably shared by a language community) are perceived by participants in a single task.

Before turning to the current experiment, let us carefully review the details of Kronmüller et al.’s (2017) original negative reference paradigm in order to fully appreciate its pragmatic features. As indicated, a participant (the addressee) is viewing three unusual-looking objects – let us call them X, Y, and Z – after being told that the speaker is viewing two objects and while assuming that the participant is seeing the same two. In the critical, experimental (what we will call the *second-object-is-named*) condition, the speaker has provided two of the three objects (X and Y) with names. What prompts a significant majority of participants to choose the least-familiar object, Z, as the intended object of “not the X” in this scenario? To start, when a participant hears “not the X,” it reveals that one of the speaker’s observable objects indeed includes the previously-named X. It also prompts the listener to create an *ad hoc* category of two objects (for seminal descriptions of *ad hoc* categories, see Barsalou, 1983), containing a second named object (Y) and an unnamed one (Z). The question is which of the two is paired with X (see Table 1 below). A listener could arguably conclude that it is more unlikely that the speaker would refer to Y as “not the X” because the speaker had previously referred to Y with a name, just as she had used X in referring to it with a name. This makes it more likely that X is paired with Z. The supposition that the speaker had Z in mind is thus optimal for resolving the reference “not the X.”

**Table 1:** A representation, from the listener’s perspective, of the two possible pairings that the speaker is viewing when saying “Not the X,” in the Kronmüller et al. (2017) paradigm.

Possibility 1		OR	Possibility 2	
X	Y		X	Z

Prior experimental pragmatic investigations into *ad hoc* pragmatic inference emerged with respect to children’s production of scalar implicatures. Scalar implicature refers to cases in which a relatively weak expression, such as *Some of the cats are black*, is thought to imply the rejection of a more informative and unsaid one (such as *All of the cats are black*) to yield the implicature *Not all of the cats are black*. Children are widely known to be less likely than adults to make this pragmatic inference (e.g., see Noveck, 2001, 2018; Katsos et al., 2016). As the reliability of the developmental effect grew and as explanations generally relied on participants’ knowledge of linguistic scales related to lexical terms, i.e., how it relies on recognizing that *All* entails *Some*,^1^ Papafragou and Tantalou (2004) investigated cases in which children can equally or more reliably exploit *ad hoc* categories in context (*Did the cow wrap the gifts? He wrapped the parrot*); these *particularized* cases (as opposed to *generalized* cases) bypass concerns about linguistic competencies, such as knowledge about and the application of lexical scales (see Grice, 1989). For an illustrative example of *ad hoc* implicature development, consider work from Stiller et al. (2015), who investigate cases in which 2- to 5-year-old children as well as adults are shown three smiley faces – one classic smiley face, a second smiley face wearing glasses, and a third wearing glasses and sporting a hat. When participants are told “My friend wears glasses,” it is at around three-and-a-half years of age that children reliably point to the smiley face wearing glasses only, even though there are two smiley faces with glasses to consider. This more precise reading of the utterance (to mean *the friend is wearing glasses but no hat*) is wholly contextual and occurs on the fly based on (1) the *ad hoc* category of three presented smiley-faces (i.e., without concerns about lexical scales), and while (2) inferring that the speaker would have said “my friend is wearing a hat” if that was indeed the friend the speaker had in mind. In fact, adult-like performance appears to emerge earlier among children in experimental situations that call on *ad hoc* implicatures when compared to those that rely on knowledge about linguistic terms and scales.

As should be clear, *ad hoc* implicatures similarly come into play in the negative reference task, whose contextual cues create an *ad hoc* category from which one can make more precise interpretations; in this case, the process begins with a negative reference. When a speaker says “not the X,” leaving two objects to be considered as a target, a listener’s interpretation (which of the two did the speaker have in mind?) depends on what he knows about prior references. In what is essentially the control condition (what we will refer to later as *the baseline*), where X is the only object with a name, listeners have no reason to favor one of the unknown objects as the speaker’s referent over the other; *Not the X* ought to lead to random responding among the options Y and Z, as has been reported. When one of the remaining objects (Y) has also been given a name, however, listeners are more likely to exclude it from consideration as a partner for X and arguably because the speaker declined to be more informative by referring to it as Y when the opportunity was there.

Building over these findings, the current investigation has two aims. One is to determine whether recognizable, conventional-looking objects affect participants’ navigation of the *ad hoc* implicature in the same way as named unconventional objects have been shown to do in this task. To anticipate, imagine that we slightly transform the original control condition so that Y remains an unconventional, unnamed object but Z is now a recognizable, conventional-looking object. Will listeners disregard the conventional-looking object and pragmatically reason their way to choose the unnamed, unconventional-looking target referent (much like they do when there is a named unconventional object) or will they consider the conventional-looking object, which is never explicitly named, as having equal status to the unnamed, unconventional object? To put it another way, to what extent does the presence of a (readily identifiable) conventional-looking object prompt participants to exclude it from consideration upon hearing “Not the X”? Assuming that participants do eliminate a conventional-looking object from consideration in such circumstances, we then ask to what extent does this sort of information compete with a case in which the other, unconventional object is indeed named. In this way, we can capture how well (unnamed) conventional-looking objects measure up to properly named unconventional ones.

This preamble sets up what follows. Below, we present the task as it was inspired by Kronmüller et al. (2017), which was conducted in Spanish, while investigating negative reference with pre-recorded materials. We monitor listeners’ moment-by-moment interpretation of negated references using a visual-world eye-tracking task. Two other critical dependent variables are participants’ final referent selections and their reaction times in making selections.

## Materials and Methods

### Participants

Participants include 48 native speakers of Spanish of which 26 were male. Their mean age was 22 and ages ranged from 18 to 32. Forty-three were undergraduate students from different faculties and five were professionals. They participated in exchange for 5,000 Chilean Pesos (approx. 8 USD).

### Design

The experiment had four conditions that were administered within subjects. Each condition was defined by the way the test trial juxtaposed two kinds of remaining objects (what we have been calling objects Y and Z), after one named unconventional object (X) had been ruled out by the speaker (through “not the X”). We essentially turned the objects Y and Z into variables, by calling them the *second* and *third* objects. That is, the presentation of each of these two remaining objects was systematically varied, based on the following: (1) we varied whether or not the Y (the second) object, which was always unconventional looking, had been previously designated with a name, and (2) we varied whether or not the Z (the third) object, which always remained unnamed, was conventional looking. Table 2 summarizes the experiment’s design and its four condition names.

**Table 2:** The design of the experiment’s four conditions, including a description of all three objects in each upon hearing the negative reference, “not the X,” which refers to the previously named unconventional object that is found in each condition.

	First object (X)	Second object (Y)	Third object (Z)
	*Always unconventional and named*	*Always unconventional (varies naming)*	*Always unnamed (varies conventionality)*
**Condition name**			
Baseline	Unconventional-named	Unconventional-unnamed	Unconventional-unnamed
Second-object-is-named (SON)	Unconventional-named	Unconventional-named	Unconventional-unnamed
Third-object-is-conventional (TOC)	Unconventional-named	Unconventional-unnamed	Conventional-unnamed
SON-vs.-TOC	Unconventional-named	Unconventional-named	Conventional-unnamed

The first condition is called “Baseline” because the negated reference of the X object leaves participants a choice between the two remaining objects (Y and Z) that are both unnamed, unconventional objects. This baseline condition is identical to the control condition in the original Kronmüller et al. (2017) paper (it equally serves as a control condition here and as a way to confirm prior results). The second-object-is-named (SON) condition refers to the case in which the negated reference of the X object leaves participants a choice between a previously named, unconventional object (Y) and an unnamed, unconventional object (Z). This condition corresponds to Kronmüller et al.’s (2017) experimental condition as described in the section “Introduction.” The third-object-is-conventional (TOC) condition leaves the participant a choice between an unnamed unconventional object (Y) and a conventional-looking (Z) object (that is never explicitly named). The third condition is new to this paradigm but conceptually identical to the second-object-is-named (SON) condition. The difference between them is that we are testing whether the recognition of conventional visual information can serve as the basis of an *ad hoc* implicature. The final – SON-vs.-TOC condition – leaves the listener a choice between a named, unconventional object (Y) and unnamed, conventional-looking object (Z). This condition, which is also new to this paradigm, forces participants to choose between two “conventionalized” objects, one (Y) that was coined conversationally against an object (Z) that is assumed to be conventionalized visually. Each participant received one of four stimulus lists created by rotating each set of objects through all four conditions, such that any individual participant saw all sets. See Figure 1 for comparable examples of a single test-trial for each condition.

### Procedure

To start, the experimenter informed participants that they would be playing a game in which their task was to identify and select target pictures based on recorded directions from a previous speaker. The pictures were presented on a computer screen and selection was made by clicking on the picture with the computer’s mouse. Participants were led to believe that the previous speaker was a naïve participant playing the “director” role in a communication game whose spontaneous speech was recorded while giving instructions to a “matcher” participant in a previous session. Critically, they were led to believe that during the previous session, the director and the matcher worked from different computer screens and were prevented from viewing each other’s screen by a divider. During the experimental session, speech was automatically triggered by the software which also recorded the identity of the object selected and the time it took participants to make the selection.

**Figure 2:**
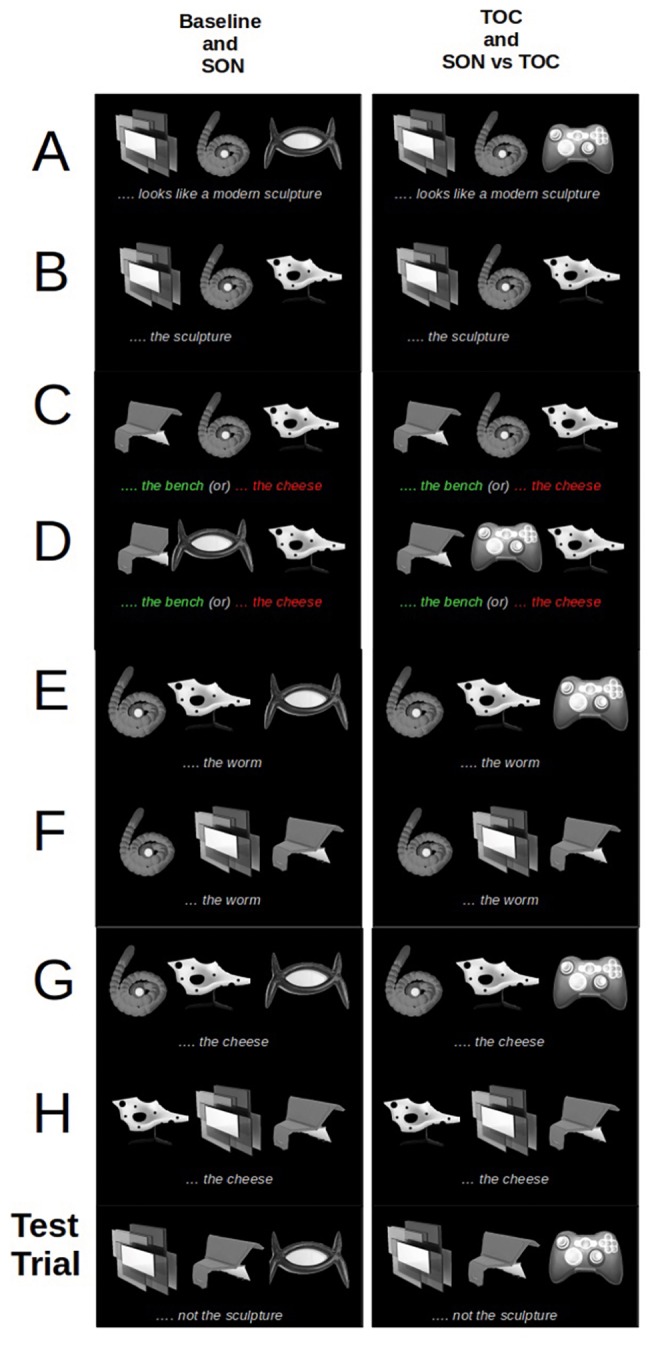
An example of a round for each condition, including all the trials, showing the objects presented and the instructions a participant heard. The column on the left shows the objects and instructions of the baseline and second-object-is-named (SON) conditions; the column on the right depicts the objects and instructions regarding the third-object-is-conventional (TOC) and the SON-vs.-TOC conditions. In general, when the objects were named for the first time, the instructions were richer; for example, with respect to the second column in row A, the instruction would be “Click on the object that looks like a modern sculpture,” whereas the second time, the instruction would be of the form “Click on the sculpture” or simply “The sculpture.” Rows A and B correspond to the grounding trials that set up the negated object (X) in the test trial (the sculpture). Rows C and D depict the objects for the grounding trials among which the second object (Y) gets named or not. When it is named (in the SON and SON-vs.-TOC conditions), the instruction concerns the object circled in green (the bench). For the baseline and TOC conditions, there is another object that is named, circled in red, but it does not appear in the test trial, leaving the second object in the eventual test trial unnamed. Rows E and F are filler trials. The trials in rows G and H along with the test trial are all part of the completion phase. The trials in the completion phase appear in a pseudo-random order so that the test trial appears as either the 7th, 8th or 9th trial, so as to mask the purpose of the study.

For presentation purposes, we conceive of a single “trial” as an event in which a participant views a display and interprets the speaker’s instruction to click on one of the displayed objects. A “round” is a collection of trials. Figure 2 shows an example of a round for each condition. The trials making up a round can be subdivided into two phases: a “grounding phase” (rows A–F) and a “completion phase.” The completion phase is designed to present a condition’s “test trial” (one of the four in Figure 1) pseudo-randomly along with two other trials (those in rows G and H) such that the test trial can appear anywhere in this phase. In other words, the completion phase ultimately *includes* the “test trial” but it could arise as the 7th, 8th, or 9th trial. This was done to mask the purpose of the experiment. We performed analyses on the test trial data only, regardless of its position in the round.

The grounding phase itself consisted of four “grounding trials,” used to set up the names for the test trial objects (lines A, B, C, and D), plus two “filler trials” where names were repeated or where other objects were referred to (lines E and F). In two of the grounding trials (A and B), a name is given to the object (X) that will later be negated in the test trial. In two other grounding trials (C and D), an unconventional object is either named [for the second-object-is-named (SON) or the SON-vs.-TOC conditions (see the utterance in green)], or is not named [for the baseline and the third-object-is-conventional (TOC) conditions (see the utterance in red, where an object *not appearing in the test trial* is given a name)]. Note that for the baseline and second-object-is-named (SON) conditions, the third object was also an unconventional object that would not be easy to name. In contrast, in the third-object-is-conventional (TOC) and SON-vs.-TOC conditions, the third object (Z) is a conventional-looking object (though, as always, it was never explicitly named).

Grounding trials were pseudorandomized, so that A, C, and E would always appear before B, D, and F. The rationale for this is to allow for a first mention to be more descriptive such as “the one that looks like a modern sculpture,” and so that the second mention is a more concise one, such as “the sculpture.” This simplification of a description is a well-known phenomenon in dialogue research (Clark and Wilkes-Gibbs, 1986).

As indicated above, the test trial was pseudo-randomly presented as one of the last three trials of a round. In each test trial, the speaker used a negative referring expression to identify the target, such as “not the sculpture” (Spanish: “no la escultura”). As can be seen in Figure 1, listeners saw three referential alternatives, the negated object (X), the second (Y), and third (Z) objects (for the sake of exposition, Figure 1 presents these three at the top, the lower left, and the lower right, respectively), with each appearing with equal frequency in the grounding phase.

One concern with the task is that the speaker’s negated descriptions may seem uncooperative, since the description would be insufficient for distinguishing between two possible alternatives. To avoid perceptions of uncooperativeness, we added additional procedures and a cover story (similar to those used in past instantiations of this experiment in the literature). Participants were told that when the instructions were recorded, the speaker and the listener viewed their displays on different computer monitors; we also led the listener to believe that the speaker saw only two of the three alternatives that the listener saw, but that the listener did not know which two. Given this setup, listeners would have no reason to find the speaker’s behavior uncooperative. In order to keep this feature salient to the participant, the experiment on occasion would request the participant to guess which was the object that the speaker was not seeing.

### Materials and Apparatus

Sets of six objects were prepared for each of the 24 rounds (144 different objects in total). For each set, five of the six were unconventional objects: one for the negated object (X), one for the second (Y) object that could potentially be named, and one for the third (Z) object when it was unconventional, plus two different filler objects. The sixth was a conventional-looking object that could serve as the third (Z) object when called for. All of the images were downloaded from the Internet and were converted to grayscale so that they could not be identified by color.

We tracked listeners’ eyes using an EyeLink 1,000 eyetracker (SR~Research). The system used a remote tabletop camera, allowing relatively free head movement. Gaze data were recorded at a sampling rate of ~500 Hz.

### Data Analysis

We analyzed participants’ proportion of selections as well as their reaction times and gaze patterns to each object. Selection data reflect the participant’s final referential commitment. Eye movements, in contrast, inform us about the interpretation process in real time. Because the main interest was the relation between the second (Y) and third (Z) objects, for all inferential statistics our dependent variable is the “log-ratio” of selecting the third (Z) object over the second (Y) object across conditions: a log ratio of zero means no preference, a positive value means preference for the third (Z) object, and a negative value points to a preference for the second (Y) object.

All *p*’s in the selection and eye-movement analyses, for subjects (p1) and items (p2), were obtained using a resampling technique. We generated a permutation scheme through which a decision was made to either keep the original labeling or change the labels for all four conditions (so no data point kept its original labeling). We built 9,999 data sets based on Monte Carlo samples for all possible arrangements of the data following our permutation scheme. For the selection data, we fit a baseline-category multinomial logistic regression (Agresti, 2002) to each of these datasets and built a null hypothesis distribution with all regression coefficients against which the original coefficient was contrasted. The proportion of coefficients from the null hypothesis distribution greater than the original constitutes the *p* for a specific contrast. We take the baseline condition as the reference group in a dummy coding scheme.

Given the complexity of analyzing eye-tracking data, mainly due to the fact that the time series is categorical, and in order to avoid arbitrary identification of time windows to perform the statistical analyses, we follow a “cluster randomization” approach as it has been previously adapted to visual world eye-tracking experiments and specifically to the original version of the task we present here (see Barr et al., 2014 for a thorough explanation of this approach). In short, the algorithm identifies periods of time during which two time series diverge. Finally, reaction times were analyzed using a mixed-effects regression, with subjects and items as random factors. We include the maximal random effect structure justified by the design and that converged, which in our case was all random effects (intercepts and slopes) but excluding their correlations (Barr et al., 2013). *p*’s are obtained using a model comparison approach. All analyses and graphics were performed using R (Bates et al., 2013).

## Results

### Selection

**Figure 3:**
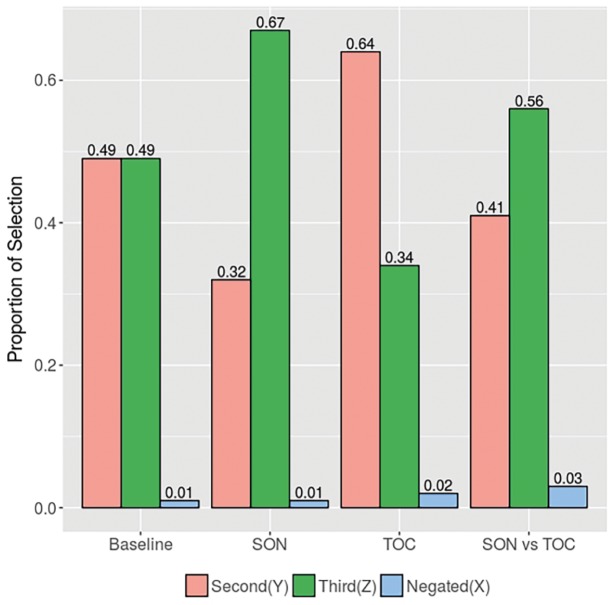
Proportion of selection of each object per condition.

Figure 3 summarizes the results from the four conditions. In the baseline condition, the proportion of selection of the second (Y) object (0.49) was equal to the selection of the third (Z) object (0.49) (log-ratio = 0). In the SON condition, in contrast, the selection of the third (Z) object (0.67) was 2.1 times higher than the proportion of selections of the second (Y) object (0.32). This log ratio (0.74) is different than the zero log ratio in the baseline condition (*p*1 < 0.001, *p*2 < 0.001). In the TOC condition, the proportion of selection of the second (Y) object (0.64) was 1.9 times higher than the third (Z) object (0.34); this log ratio (−0.63) is also significantly different than the ratio in the baseline condition (*p*1 < 0.001, *p*2 < 0.001). Finally, in the SON-vs.-TOC condition, the proportion of selection of the third (Z) object (0.56) was 1.4 times higher than the second (Y) object (0.41); this log ratio (0.31) is not statistically different from the log ratio in the baseline condition (*p*1 = 0.148, *p*2 = 0.115).

#### Individual Differences

**Figure 4:**
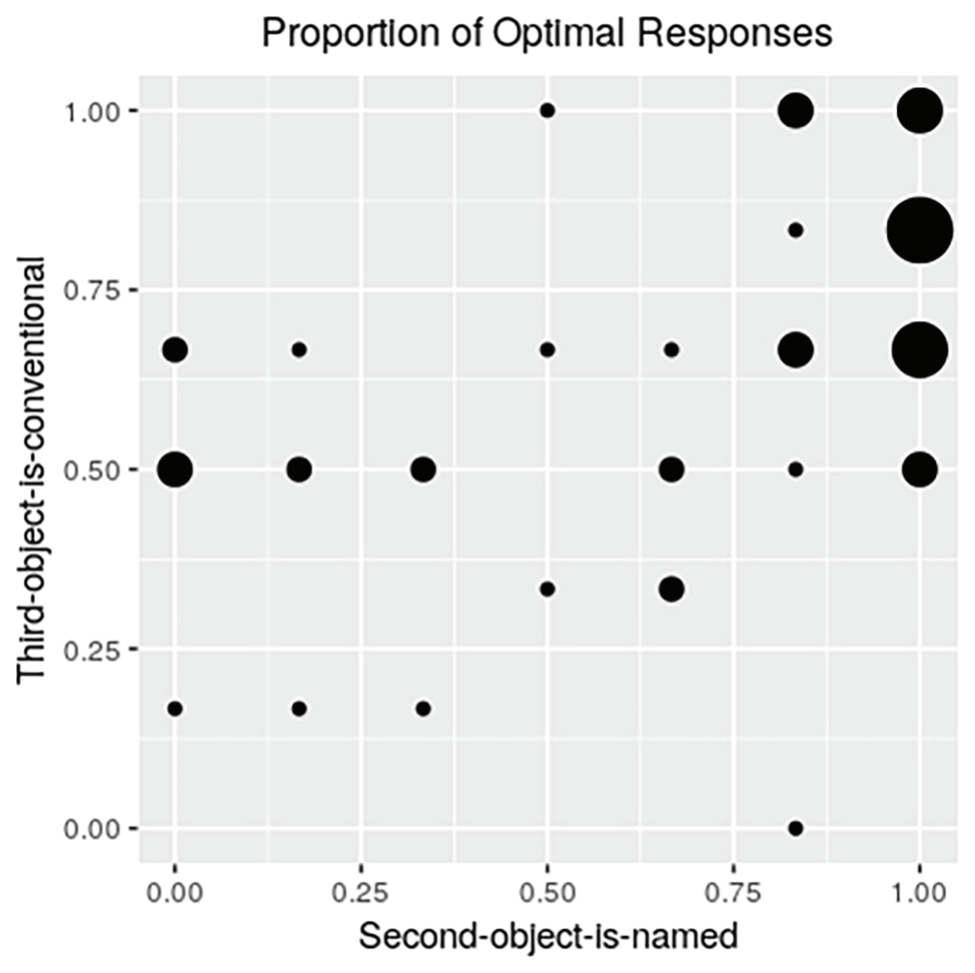
Individual differences: proportion of optimal responses in the second-object-is-named (SON) and the third-object-is-conventional (TOC) conditions. The size of each dot on the grid represents the relative number of participants at that coordinate with respect to the proportion of optimal responses provided in the SON condition (x axis) and the proportion of optimal responses provided in the TOC condition (y axis).

In order to observed individual tendencies, we present the proportion of “optimal” responses. An optimal response is defined as the speaker’s likely intended object post-negative-reference (after “not the X”), based on conversational considerations. In the case of the SON condition, this means selecting the third (Z) object (which is the remaining unnamed and unconventional object in this condition). In the case of the TOC condition, in contrast, the optimal alternative is the second (Y) object (which is the remaining unnamed and unconventional object, for this condition). In both of these cases, the optimal response is the one for which there is the least information. Figure 4 presents the proportion of selections of the optimal alternative. Each dot on the grid represents the relative number of participants at that coordinate with respect to the proportion of optimal responses provided in the SON condition (x axis) and the proportion of optimal responses provided in the TOC condition (y axis). The participants in the upper right corner of the graph were consistently optimal in their responding across the two conditions. The relatively empty lower left corner of the graph represents those participants who choose both the named object in the SON condition and the conventional-looking object in the TOC condition. By visual inspection, it can be observed that there are, roughly, two groups. Most of the participants tend to make optimal responses, but there is a smaller group (on the left half of the graph) that systematically gives non-optimal responses in the SON condition, by selecting the object (Y) that had in fact been given a name, while being somewhat indifferent with respect to the TOC condition, by selecting the conventional object (Z) about half the time. The two right-most columns reveal that the SON condition provides optimal responses more consistently than the TOC condition.

### Reaction Times

Here, we present the time it took – in milliseconds – to select an object upon hearing the negative reference, beginning from the offset of the noun (for example, from the offset of “sculpture” in “not the sculpture”). Reaction times show an interesting pattern. Reaction times are longer in the baseline condition (*M* = 3,518; SD = 1,403) compared to the SON condition (*M* = 2,530; SD = 805) [*χ*^2^(1) = 38.523; *p* < 0.001]. Likewise, reaction times are longer in the baseline condition when compared to those in the SON-vs.-TOC condition (*M* = 2,888; SD = 1,105) [*χ*^2^(1) = 16.201; *p* < 0.001]. In contrast, reaction times in the TOC condition (*M* = 3,302; SD = 1,305) were not statistically different from those in the baseline condition [*χ*^2^(1) = 2.061; *p* = 0.151]. Means and standard deviations were computed aggregating by subjects.

### Eye Movements

**Figure 5:**
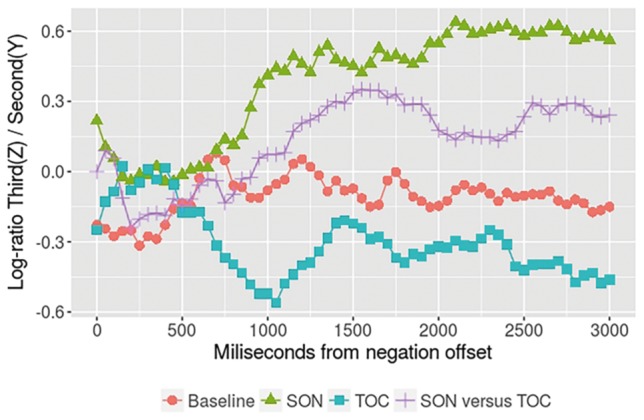
Log ratio across time from zero milliseconds after the offset of the noun in the negated reference up to 3,000 ms. A line around zero means no preference. A positive value reflects a preference for third object (Z), and a negative value reflects a preference for the second object (Y). Each line depicts a condition.

Figure 5 shows the preferences for either the second (Y) or third (Z) objects from the offset of the referring expression up to 3,000 ms. As can be observed, the second-object-is-named (SON) condition reveals that there is an early preference for the third (Z) object (which is unnamed and unconventional looking) that starts at 700 ms and is sustained until the end of the time window. In the TOC condition, there is an opposing pattern, with an early preference toward the second (Y) object (which is unnamed). However, in this case, this preference does not grow monotonically until the end of the window; after their initial quick decision, participants appear to hesitate by looking back and forth between the two remaining objects. In the SON-vs.-TOC condition, there is an early preference for the (conventional looking) third (Z) object, but less than in the SON condition; much like in the TOC condition, their early preference is followed by some apparent indecision. Finally, as expected, the baseline condition is around zero throughout the entire time window.

These observations are corroborated by statistical analyses: two clusters can be identified when comparing the baseline condition against all others. For the SON condition, there is a reliable cluster that starts at 900 ms up to the end of the time window (*p*1 < 0.001, *p*2 < 0.001). For the TOC, there is a reliable cluster between 650 and 1,300 ms (*p*1 = 0.036, *p*2 = 0.042). Finally, even when there is a numerical difference between the SON-vs.-TOC and the baseline conditions, starting approximately at 1,000 ms up to 2,200 ms, there is no reliable cluster identified by the cluster randomization algorithm.

## Discussion

Prior work with a well-established negative reference task (Kronmüller et al.’s, 2017) has shown that, post-negative reference, participants reliably look past a previously named object in order to choose an unnamed unconventional object. This effect was replicated here through the second-object-is-named (SON) condition. We argue that participants hypothesize that, if an object can be readily referred to with a name, a speaker would have done so and that, if a speaker does not do such referring, it is taken as a clue that the negative reference is more likely referring to the unnamed unconventional-looking object. The present work extends this finding by investigating a highly similar situation in which a conventional-looking object that is never named (for example, the object that resembles a gamepad in our figures) is juxtaposed with an unnamed unconventional object. The question that we asked in this work was, would participants similarly look past the conventional object and choose the unnamed, nondescript alternative? The paradigm also allowed for a situation in which the named unconventional object could be set against the unnamed conventional-looking object.

The results from the third-object-is-conventional (TOC) condition indeed show that participants give optimal responses at rates that are comparable to those in the second-object-is-named (SON) condition. That said, the effect linked to the third-object-is-conventional (TOC) condition does not appear to be as a strong. This can be inferred from three results. The first is that participants’ eye-tracking patterns toward the remaining unnamed unconventional object (Y) appear less resolute in the third-object-is-conventional (TOC) condition than they are to the unnamed unconventional object (Z) in the second-object-is-named (SON) condition. Participants do immediately focus on the most nondescript object in the third-object-is-conventional (TOC) condition, but they also reveal some hesitation soon afterward. This is unlike participants’ reactions in the second-object-is-named (SON) condition in which they maintain their focus on the remaining unnamed unconventional object (Z) and in an increasingly monotonic fashion. The individual difference data in Figure 4 provide further supporting evidence showing that the SON condition provides more resolute decision making than the TOC condition. There, one sees 100% or near 100% optimal choice making for a majority of the participants (the two rightmost columns of Figure 4), which reflects optimal performance on the second-object-is-named (SON) condition, while optimal performance for the third-object-is-conventional (TOC) condition is less common for the top two rows of Figure 4.

The second result is that reaction times in the third-object-is-conventional (TOC) condition were as slow as in the baseline conditions. In contrast, in the second-object-is-named (SON) condition, reaction times were relatively fast. And, finally, the third result is that when the two sorts of cases are forced to compete in the SON-vs.-TOC condition, one can detect that there is a slight (though not statistically reliable) tendency to favor the unnamed conventional object (Z) as the participant’s choice selection. Both in terms of selections and in terms of eye-tracking patterns, listeners tend to look past the named object and to choose the unnamed conventional-looking object as the speaker’s referent. This summary, of course, refers to overall preferences for this particular task. Clearly, there are many participants who do not use the speaker’s cues to make what we refer to as the optimal response. It is also important to keep in mind that the conventional-looking object is never given a mutually manifest name. It will be useful for future work to determine the extent to which a *named*, conventional-looking object determines performance on this task. The aim of that work will be to determine whether the effects of naming and conventional appearance are additive.

The regularity of these results is quite remarkable once one considers that participants are simply receiving a negative sentence in the form of “not the X,” which leaves two options and much else to be determined. To come up with what this paradigm considers to be the optimal response, listeners are arguably reasoning that the speaker could have made a more direct and informative, i.e., more facilitative, utterance [e.g., *(Click on) the Y object*] but did not. In light of this, the addressee is justified in assuming that the object in such an unspoken – and potentially more informative utterance – is *not* the one that the speaker had intended to point out. This leads the listener to conclude that the speaker did not intend to refer the *Y* object in the second-object-is-named (SON) condition; likewise, it leads a listener to the conclusion that the speaker did not intend to refer to the *Z* object in the third-object-is-conventional (TOC) condition.

### *Ad hoc* Implicature

The current findings show the extent to which pragmatic reasoning need not rely on linguistic features. As we noted in the section “Introduction,” we argue that optimal choices result from a particularized or *ad hoc* implicature in which listeners consider the contents of a two member category created by the negative reference; more specifically, participants look past the object that has the potential to be readily informative because the speaker did not mention it. These findings edify our understanding of *ad hoc* implicature-making in three important ways.

The first is that a listener’s *ad hoc* pragmatic reasoning here leads to an optimal reference at above-chance levels in the SON and TOC conditions even though the speaker is assumed to be viewing just two of the three objects in front of the listener. To make a non-random reference, the listener actually needs to *infer* which objects appear on the speaker’s screen, based on what was said (or observed). In other words, the listener needs to generate a speaker’s epistemic state in order to justify the optimal choice. This is more complicated than what occurs in classic *ad hoc* implicature tasks, in which a speaker refers to a category of, say, three similar drawings and the speaker is assumed to have the same, stable view as the listener. Nevertheless, the relatively reliable results reported here indicate that listeners use a procedure that is similar to those found with other *ad hoc* implicatures, in which participants consider what could have been said but was not.

The second is that optimal responding need not be determined uniquely by prior actions taken by the speaker. The mere presence of a conventional looking object, one that occasionally and namelessly appears across a round (as is the case for the conventional looking object in the TOC condition), also encourages listeners to assume that the negative reference leads to an optimal response, i.e., to choose the most non-descript object. This shows how *ad hoc* implicature-making is opportunistic; a listener will seek out any sort of evidence in an effort to identify an alternative that can make distinctions with regard to informativeness. When the salient conventional-looking object is not referred to in the third-object-is-conventional (TOC) condition, it is presumably a clue to the participant that it is not on the speaker’s screen.

Finally, this is the only paradigm we are aware of in which *ad hoc* procedures operate reliably in a wholly negative space. Participants begin their calculation through a negative reference and then disprefer one object out of the remaining two based on their interlocutors’ conversational history (in the SON condition) or on the salience of a potentially nameable object (in the TOC condition). Optimal performance is not based on contrasts between highly similar objects (such as smiley faces) or on the categories that the objects can spontaneously belong to. Overall, participants’ ability to find optimal responses in this difficult context is impressive.

### Conclusions

The investigation here is exemplary of the kind of work that is needed to better understand the role played by conventionalizations in language as they are employed in utterance understanding. There is much else left to do. Other questions that experiments in this genre can answer are the following: are there indeed isolable procedures linked to conventional-looking objects? One can also ask how does performance on this task develop? These and other experimental pragmatic questions can be addressed by turning one’s attention to conventionalized meanings in dialogue and to conventional-looking objects.

In conclusion, a negated reference in the current paradigm forces a listener to rely almost exclusively on contextual information in order to infer communicative intentions. We show how the negative reference *Not the X* triggers the creation of an *ad hoc* category and a pragmatic process through which the listener needs to evaluate two alternatives, with the optimal response amounting to recognizing which object could have been identified in an informative way but was not. Ultimately, listeners needed to identify the least mutually-recognizable object of two in a task that started with a negative expression. It appears that unconventional-looking objects having temporarily shared names carry slightly more weight than conventional-looking objects that are never explicitly referred to.

## Ethics Statement

This study was carried out in accordance with the recommendations of ethical guidelines of the Comité Asesor de Bioética del Fondo Nacional de Desarrollo Científico y Tecnológico de Chile. All subjects gave written informed consent in accordance with the Declaration of Helsinki. The protocol was approved by the Comité Asesor de Bioética del Fondo Nacional de Desarrollo Científico y Tecnológico de Chile.

## Author Contributions

This represents the authors’ third joint project. EK and IN conceived the current study. EK designed it, ran it and analyzed the results. The joy in appreciating the findings and writing up the paper was equally distributed.

### Conflict of Interest Statement

The authors declare that the research was conducted in the absence of any commercial or financial relationships that could be construed as a potential conflict of interest.

## Footnotes

^1^For full fledged linguistically-based account of this developmental effect (one that relies on scales of informativity), see Barner et al. (2011).
